# Tri-Layer Core–Shell Fibers from Coaxial Electrospinning for a Modified Release of Metronidazole

**DOI:** 10.3390/pharmaceutics15112561

**Published:** 2023-10-31

**Authors:** Ying Wang, Lin Liu, Yuanjie Zhu, Liangzhe Wang, Deng-Guang Yu, Li-ying Liu

**Affiliations:** 1 School of Materials and Chemistry, University of Shanghai for Science and Technology, Shanghai 200093, China; 212203105@st.usst.edu.cn; 2 Naval Medical Center, Naval Medical University, Shanghai 200433, China; earnestliu@126.com (L.L.); 13918338960@126.com (Y.Z.); lzwang@hotmail.com (L.W.)

**Keywords:** emulsion electrospinning, coaxial electrospinning, modified release, nanostructure, drug delivery, polymeric hybrids

## Abstract

Polymers are the backbone of drug delivery. Electrospinning has greatly enriched the strategies that have been explored for developing novel drug delivery systems using polymers during the past two decades. In this study, four different kinds of polymers, i.e., the water-soluble polymer poly (vinyl alcohol) (PVA), the insoluble polymer poly(ε-caprolactone) (PCL), the insoluble polymer Eudragit RL100 (ERL100) and the pH-sensitive polymer Eudragit S100 (ES100) were successfully converted into types of tri-layer tri-polymer core–shell fibers through bi-fluid coaxial electrospinning. During the coaxial process, the model drug metronidazole (MTD) was loaded into the shell working fluid, which was an emulsion. The micro-formation mechanism of the tri-layer core–shell fibers from the coaxial emulsion electrospinning was proposed. Scanning electron microscope and transmission electron microscope evaluations verified the linear morphology of the resultant fibers and their obvious tri-layer multiple-chamber structures. X-ray diffraction and Fourier transform infrared spectroscopy measurements demonstrated that the drug MTD presented in the fibers in an amorphous state and was compatible with the three polymeric matrices. In vitro dissolution tests verified that the three kinds of polymer could act in a synergistic manner for a prolonged sustained-release profile of MTD in the gut. The drug controlled-release mechanisms were suggested in detail. The protocols reported here pioneer a new route for creating a tri-layer core–shell structure from both aqueous and organic solvents, and a new strategy for developing advanced drug delivery systems with sophisticated drug controlled-release profiles.

## 1. Introduction

Because of their special physiological environment and advantages, such as mucosa that promotes drug absorption, low proteolytic activity, long residence time, etc., specific areas of the gut are particularly suitable for some special diseases and are targeted organs for drug therapy [1,2]. Enterocolitis is a type of inflammation of several sections of the gut caused by bacteria, viruses, fungi, and parasites. It is caused by various reasons, mostly in people with unclean diets. The course of the disease is chronic, extending for several years to more than ten years, especially for ulcerative colitis, which is a very complex disease at any age, but the common age of onset is generally 20–50 years old. In general there is no significant difference between women and men [3,4]. Severe gut cancer may be induced, and according to surveys, the quantity of patients who die from this disease is gradually increasing every year [5].

Since the 1990s, drug delivery systems for specific regions of the gut have received increasing attention [6,7,8,9] because they are very effective in the localized treatment of several intestinal diseases [10,11] (e.g., ulcerative colitis [12,13] and topical cancer [14]). There are many kinds of drug controlled-release profiles for treating gut diseases, such as PH-dependent, time-delayed release, and flora triggering [15,16,17,18]. Coban et al. [19] reported the preparation of drug-loaded nanofibers of PH-sensitive Eudragit L100 (EL100) for drug delivery to a specific region in the intestine, which were loaded with the niclosamide-hydroxypropyl-beta-cyclodextrin (HP-βCD) complex through blending electrospinning. Ding et al. [20] used triaxial electrospinning to prepare ES100 core-shell nanofibers loaded with aspirin. Due to their pH sensitivity, the release of drugs in gastric juice was reduced, and the gastric mucosa was protected. To date, a large number of drugs have been reported to be delivered to specific regions of the intestine for targeted release, and often with a prolonged drug-release effect. Turanli et al. [21] obtained budesonide-supported controlled-release nanofibers in a specific region of the intestine by using pH-sensitive anionic Eudragit S100 (ES100) and time-dependent cationic Eudragit RL100 (ERL100) polymers in various proportions. Akhgari et al. [22] also used ES100 and ERS100 polymers as carriers for preparing electrospun indomethacin-loaded nanofibers for specific drug delivery. Patriota et al. [23] developed a new sort of spherical Eudragit-coated nanoparticle with the combined usage of Eudragit and chitosan (CS). These reports obviously suggested that Eudragit series polymers are popular for delivering a drug to the specific areas of the gut regardless of their formats of monolithic nanocomposites, core–shell nanofibers or particles.

Fibrous carriers have their potential applications in many areas, such as biomedical, filtration, sensing, and photovoltaic, especially in biomedical applications [24,25,26,27,28,29,30,31,32,33]. Electrospinning is a well-known method to prepare polymer fibers from nano to micron scale in a straightforward manner [34,35,36]. Accompanied by and combined with electrosprayed particles [37,38,39,40,41,42], the possible functional applications of electrospun nanofibers are continuously expanding [43,44,45,46]. Among them, electrospun nanofibers for enhancing the dissolution rate of poorly water-soluble drugs and providing a certain drug controlled-release profile are hot topics [47,48,49]. Meanwhile, by adjusting the working parameters (system parameters, e.g., polymer type, molecular weight, solution conductivity, etc., process parameters, e.g., power supply type, collector type, spinneret type, etc., and environmental parameters, e.g., humidity, temperature, etc.), the morphology, inner structure, size, and organization patterns of the resultant fibers can be easily manipulated for adjusting the drug dissolution and release behaviors in bulk solutions [50,51,52,53].

Although a single-fluid process, emulsion electrospinning is able to create core-shell structures [54,55]. In an emulsion, the drug is usually dissolved in the aqueous phase, and is then dispersed in the oil phase along with a suitable surfactant and a polymer solution of an organic solvent (or organic solvent mixture). When an emulsion is exploited as a working fluid to experience the electrospinning process, the oil phase evaporates faster than the aqueous phase, which results in a higher viscosity of the oil phase. As the aqueous phase moves towards the interior of the working fluid jets, the oil phase moves towards the edges of the fluid jets due to the viscosity gradient. Thus, the electrical stretching and solvent evaporation induces the demulsification process. In turn, nanofibers with a core–shell structure are formed and most of the drug in the emulsions is encapsulated in the core section [56,57]. This core–shell arrangement can effectively eliminate the negative initial drug burst-release effect occurring in many medicated nanomaterials [58,59,60,61]. Abdul Hameed et al. [62] blended poly (vinyl alcohol) (PVA) with chitosan, modified starch and modified cellulose and successfully prepared core–shell nanofibers loaded with cephalexin through emulsion electrospinning. Zhan et al. [63] used emulsion electrospinning to prepare waterproof PVA/poly (acrylic acid) (PAA) fibers loaded with tangeretin. Norouzi et al. [64] prepared core–shell nanofibers of sodium alginate/polycaprolactone through typical water-in-oil emulsion electrospinning. Although these single-fluid emulsion electrospinning processes concurred in the preparation of core–shell nanostructures, no reports investigate the possibility of fabricating tri-layer core–shell nanofibers from the double-fluid coaxial emulsion electrospinning process.

Traditionally, double-fluid coaxial electrospinning simultaneously treats two different polymeric solutions to fabricate core–shell nanofibers, where one polymer is encapsulated by the other [65,66,67,68,69,70]. It is common sense that the shell solution must be electrospinnable for successful coaxial preparation, whereas the core solution can be electrospinnable or unelectrospinnable [71,72,73,74]. Thus, compared with single-fluid electrospinning, coaxial electrospinning is able to provide more strategies for tailoring components, compositions, and spatial distributions of active ingredients within the nanofibers, and in turn is more powerful for developing novel functional medical fibrous products [75]. Subsequently, modified coaxial electrospinning further expands the capability of electrospinning in creating new types of nanofibers through the co-treatments of spinnable or unspinnable solutions, solvents, emulsions, nano suspensions and even slurries.

**Figure 1:**
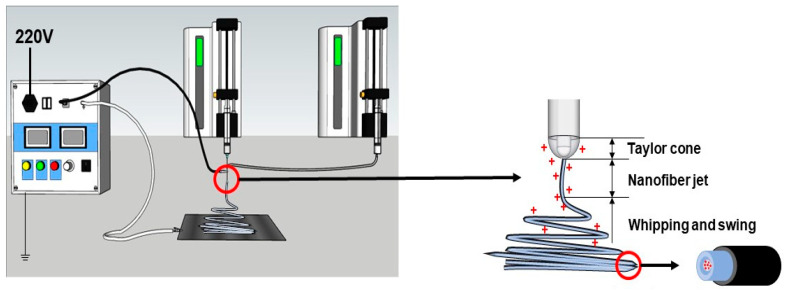
Schematic diagram of preparation of drug-loaded fibers with tertiary core–sheath structure by coaxial electrospinning using emulsion as the core.

In the present investigation, a new coaxial electrospinning process was developed with an emulsion as the core working fluid for producing tri-layer core–shell nanofibers. Metronidazole (MTD) was used as the model drug. Metronidazole, a poorly water-soluble organic compound, is mainly used as an antibiotic and antigenic insect agent. It can be used to treat colitis caused by antibiotics, and has a good effect on anti-anaerobic protozoa and anti-anaerobic bacteria [76,77,78]. A combination of two Eudragit polymers was designed as the shell section, i.e., the pH-sensitive ES100 and ERL100, to protect the drug from being prematurely dissolved in stomach acid. ES100 shows an anionic pH-dependent property, has good hydrophobic properties under acidic conditions, and has an excellent filament-forming property. Under alkaline conditions, it dissolves in water and gradually releases the drug [79]. ERL100 is cationic time-dependent and exhibits non-toxicity, high mucosal adhesion, and drug sustained-release properties [80]. The core working fluid contained Poly(ε-caprolactone) (PCL) in the oil phase, poly(vinyl alcohol) (PVA) in the water phase, and Pluronic^®^ F-127 (PF-127, a nonionic surfactant) as an emulsifier. PCL is a polymer with good biocompatibility and good biodegradability, which can be used as a cell-growth support material and can be completely degraded in 6–12 months in the natural environment [81,82]. PVA is a biocompatible, biodegradable, highly flexible and non-toxic polymer that is soluble in water and commonly used as the aqueous phase in emulsion electrospinning. Meanwhile, by varying the ratio of the two polymers in the shell fluid, their effects on the drug controlled-release profiles were also investigated. A schematic of the preparation of drug-encapsulated fibers with a tri-layer core–sheath structure from a double-fluid coaxial process is diagrammed in Figure 1.

## 2. Materials and Methods

### 2.1. Materials

Eudragit^®^ S100 (ES100, *M*_w_ = 135,000) was purchased from Röhm GmbH & Co. KG (Darmstadt, Germany). Eudragit^®^ RL100 (ERL100, *M*_w_ = 135,000) was bought from Shanghai Chineway Pharma., Tech., Co., Ltd. (Shanghai, China). Poly(ε-caprolactone) (PCL, *M*_n_ = 80,000) was obtained from Sigma-Aldrich Co., Ltd. (Shanghai, China). Poly(vinyl alcohol) (PVA, *M*_w_ = 89,000–98,000) and Metronidazole (MTD, purity of 99%, *M*_w_ = 171.15) were purchased from Shanghai Macklin Biochemical Co., Ltd. (Shanghai, China). Pluronic^®^ F-127 (PF-127) was obtained from Sigma-Aldrich Co., Ltd. (Shanghai, China). Span-80 (SP-80) was bought from Sinopharm Chemical Reagent Co., Ltd. (Shanghai, China). Anhydrous ethanol,N,N-dimethylformamide (DMF), dichloromethane (DCM), and methanol were purchased from Sinopharm Chemical Reagent Co., Ltd. (Shanghai, China). Water is deionized. All chemicals are of analytical grade.

### 2.2. Electrospinning Process

#### 2.2.1. Preparation of Core Emulsions

First, 1.0 g PCL was dissolved in 10 mL DCM/methanol (8:2) mixture as the oil phase. Then, 0.8 g of PVA was completely dissolved in 10 mL of water as the aqueous phase in a water bath at 80 °C and 600 r/min and then cooled to room temperature. After that, 0.2 g emulsifier PF-127 was added. The oil phase and the water phase were stirred for 24 h. The emulsion was prepared by mixing the oil phase and water phase in a ratio of 1:1. An amount of 0.4 g MTD was weighed and placed into the emulsion and was stirred for 24 h to form a stable emulsion with 2% (*w*/*v*) MTD.

#### 2.2.2. Preparation of Drug-Loaded Fibers by Coaxial Electrospinning

**Figure 2:**
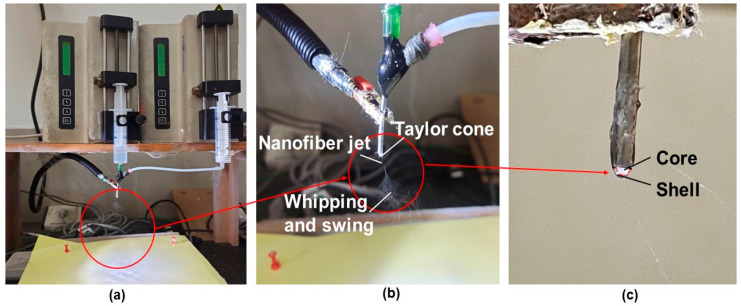
Actual spinning diagram: (**a**) Overall image of spinning; (**b**) Needle point magnification diagram of spinning process; (**c**) Core–sheath difference image (white for core fluid, transparent for sheath fluid), an enlarged image of the red circles in (**a**,**b**).

Four types of ES100 and ERL100 (ESR) blending solutions with various ratios (ES100:ERL100 was 100:0, 95:5, 90:10, and 80:20, respectively) in a DCM/methanol (8:2) solvent mixture were prepared as the sheath working fluid. The experimental system consisted of a high-voltage power supply (purchased from BMEI Limited, Beijing, China), two syringe pumps (KDS 100, purchased from KD Scientific, Holliston, MA, USA), and the homemade spinneret and fibrous collector. During the coaxial electrospinning process, a high voltage of 12 kV was applied through the tip of the spinneret, which had an outer diameter of 2.0 mm. The resultant fibers were collected on the homemade collector (a piece of aluminum foil covering a hard cardboard base and grounded), which was 10 cm away from the tip of spinneret. The shell fluid flow rate was maintained at 2.0 mL/h and core fluid flow rate was maintained at 0.5 mL/h. The collected nanofibers were always stored in a drying oven. All the experiments were carried out under environmental conditions. The actual spinning processes are included in Figure 2. Specific working parameters are shown in Table 1.

### 2.3. Characterization of Fiber

#### 2.3.1. Emulsion Stability Observation

A microscope with a camera (SK2009HDMI-TH3, Guangzhou, China) was utilized to observe the images of emulsions which had been stirred overnight. Several droplets of the emulsion were placed and spread on a microscope slide and were recorded under a magnification of 40×. The size of the emulsion droplets was estimated by ImageJ software (V.1.8.0).

#### 2.3.2. Morphology and Structure of Fibers

The surface morphologies of the fibers were observed by a scanning electron microscope (SEM, FEI Quanta 200SEM). Prior to observation, the fibers were sputter-coated with Au for surface conductivity. The diameters of fibers were assessed based on the SEM micrographs. The measurements of diameters and diameter distributions were achieved through the ImageJ software.

The interior structure of the fibers F2 was achieved through field emission transmission electron microscopy (FE-TEM, JEM2100F, JEOL, Tokyo, Japan) under an applied electron voltage of 200 kV.

#### 2.3.3. Physical States and Compatibility between the Fibrous Components

XRD measurements were carried out at room temperature using an X-ray diffraction system (XRD, Bruker-AXS, Karlsruhe, Germany) with a graphite monochromator for Cu-Kα radiation. The radiation source was operated at a voltage of 45 kV, a current of 40 mA, a scanning speed of 2 °/min, and a 2θ range from 5° to 40°.

The raw materials and their electrospun fibers were detected using Fourier transform infrared spectroscopy (FTIR, Spectrum 100, Billerica, MA, USA). All spectra were obtained at room temperature, and the scan time was set to 8 times for each sample, all in the range of 450–4000 cm^−1^ with a resolution of 2 cm^−1^.

#### 2.3.4. Water Contact Angle Test (WCA)

In order to study the water absorption and wettability of the fiber surface, the water contact angles of F1–F4 fibrous membranes were determined using the water droplet method. The water contact angle was measured using a contact angle goniometer (WCA, JY-82B Kruss DSA, Kruss, Germany) which is equipped with a digital camera. Hydrochloric acid solution (HBS, pH = 1.2) was dropped on the electrospun fibrous film through a micro syringe. Tests were performed at room temperature with at least 6 measurements per film and the mean and standard deviation were reported.

#### 2.3.5. Mechanical Properties Test (Stretch Test)

The mechanical properties of the fibrous membranes were tested by a 2.5 kN universal material testing machine (YG006, Shanghai, China). The fibrous strips were cut into a size of 20 × 50 mm^2^, and their thicknesses were recorded. Each strip was tested five times, and the obtained data were compiled and plotted as a stress–strain curve.

### 2.4. In Vitro Dissolution Test

In the in vitro dissolution tests, HBS (pH = 1.2) was utilized to simulate gastric juice, and the phosphate buffered solution (PBS, pH = 7.4) was exploited to simulate intestinal fluid. The HBS was prepared as follows: a certain amount of deionized water was taken, 2.0 g sodium chloride (AR) was added, then 7 mL concentrated hydrochloric acid was added, and later the containers were filled with water to a constant volume of 1000 mL. The PBS was prepared by dissolving 10 g PBS tablets in 1000 mL deionized water, stirring fully for reserve use.

The absorbances of 7.5–17.5 µg/mL MTD in PBS and HBS were measured by UV-visible light spectrophotometer, and the maximum absorption wavelength (λ_max_) was determined to be 318 nm and 277 nm, respectively.

In order to detect the drug release profiles from the fibers, in vitro dissolution tests were carried out. According to Method II of Chinese Pharmacopoeia (2020 Ed.), the in vitro dissolution experiments were carried out in a constant temperature oscillator (THZ312, JingHong Instrument Co., Ltd., Shanghai, China). The temperature was set at 37 °C. The rotational speed was set to 60 r/min. About 100 mg drug-loaded fibers were weighed, and were placed into the preheated 450 mL HBS, 4 mL solution was drawn at a specific time point, and the same volume of fresh preheating media was added. After 2 h, the fibers were transferred to a preheated 450 mL PBS, the drug release experiments were continued for 10 h, and the same volume of fresh media was added after sampling at a specific time point. The absorbance of the obtained sample solutions was measured by UV-visible spectrophotometer. The MTD absorbances were measured at a wavelength of λ = 277 nm and λ = 318 nm for HBS and PBS solutions, respectively. All experiments were repeated at least in triplicate. After 12 h of drug release, the fibrous membranes were removed and air-dried overnight, and then they were observed using SEM. The cumulative release (Q) can be calculated by the following formula [83]:

(1) $$Q=\frac{c_{n}{\;V}_{0}+V\sum_{i=1}^{n-1}c_{i}}{m}\times 100\%\;$$

where c_i_ and c_n_ are the drug concentrations at time points i and n, m is the theoretical mass of MTD in the fibers, V is the volume of solution removed for each measurement, and V_0_ is the volume of the release medium.

### 2.5. Drug-Release Kinetic Studies

The drug-release kinetics and mechanism are very important for developing new types of drug delivery systems. To investigate the release kinetics and mechanism of MTD, the cumulative release data of MTD from F1–F4 fibrous membranes were treated using different kinetic models [84,85], which are listed as follows (Equations (2)–(5)):

(2) $$\mathrm{Zero}-\mathrm{order}\;\mathrm{kinetics}\;\mathrm{model}:\;\;\;\;\;\;Q=k_{0}\;t$$

(3) $$\mathrm{First}-\mathrm{order}\;\mathrm{kinetics}\;\mathrm{model}:\;\;\;\;\;\;Q=1-e^{tk_{1}}$$

(4) $$\mathrm{Higuchi}\;\mathrm{kinetics}\;\mathrm{model}:\;\;\;\;\;\;Q=k_{H}{\;t}^{\frac{1}{2}}$$

(5) $$\mathrm{Ritger}–\mathrm{Peppas}\;\mathrm{kinetics}\;\mathrm{model}:\;\;\;\;\;\;Q={\mathrm{kt}}^{n}$$

In the formula, Q is the release percentage, t is the release time, k_0_, k_1_, k_H_, k is the release constant, and n is the parameter characterizing the release behaviors of drug molecules. In the Ritger–Peppas kinetic model, the diffusion index, n, is an important index to indicate the mechanism of drug release, usually between 0 and 1.

## 3. Results and Discussion

### 3.1. Emulsion Microstructure and Size

**Figure 3:**
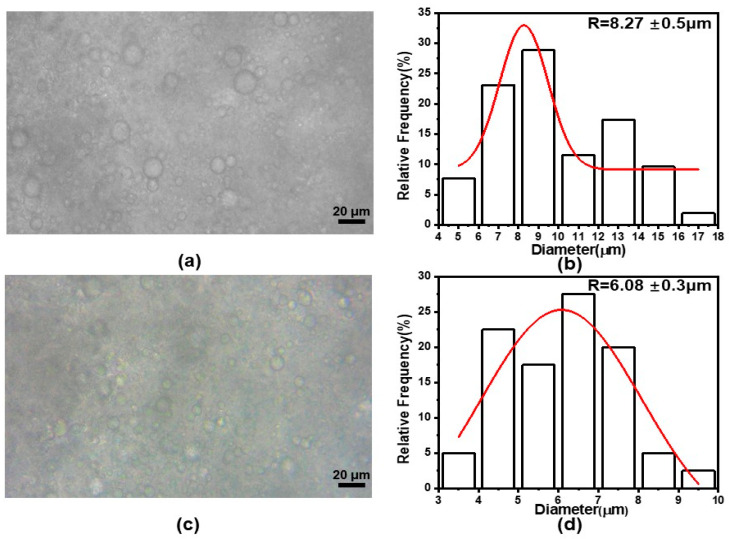
Microstructure and size of the emulsion droplets: (**a**,**c**) Optical microscope images after 12 h and 24 h agitation, respectively; (**b**,**d**) Average diameters and their size distribution of emulsion droplets after 12 h and 24 h agitation, respectively.

The stability as well as the homogeneity of the emulsion can be understood by visualizing the structure and dimension of the emulsion droplets. Figure 3 illustrates the optical microscope images of the emulsions along with the mean diameter distribution. As shown in Figure 3a,b, the dispersion of the emulsion droplets (after 12 h stirring) was not very homogeneous, but most of the emulsion droplets were of a normal spherical form with few non-spherical droplets. The average diameter of the emulsion droplets was about 8.27 ± 0.50 μm. Figure 3c,d show the optical microscope images as well as the diameter distribution after stirring for 24 h. Compared with those with 12 h of stirring, the emulsion droplets were more uniform and the average diameter was smaller, about 6.07 ± 0.30 μm. These are no observed emulsion delamination phenomena in the images, which indicates that the emulsion was well stabilized and was more conducive to electrospinning. Meanwhile, it is suggested that the longer the stirring time was applied, the more uniform size distribution the emulsion droplets had, and the smaller the average diameter of the droplets formed.

### 3.2. Morphology, Structure and Size Analysis of Electrospun Fibers and Their Formation Mechanism

**Figure 4:**
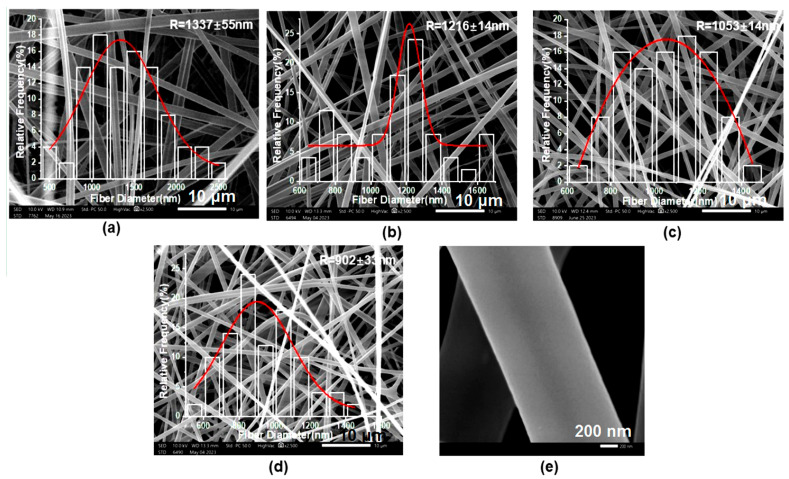
SEM images and diameter distributions of the drug-loaded fibers: (**a**–**d**) fibers F1, F2, F3, and F4, respectively, and (**e**) enlarged SEM images of fibers F2 under a larger magnification.

Figure 4 displays the SEM images and average diameter distributions of the four kinds of drug-loaded fibers. From these figures, it is clear that all these fibers showed linear morphology without beads or spindles. The average diameters of F1–F4 were 1337 ± 55 nm, 1216 ± 14 nm, 1053 ± 14 nm, and 902 ± 33 nm, respectively. As the ratio of ERL100 increased in the shell section from fibers F1 to F4, their average diameters decreased simultaneously. This has a close relationship with the interaction between anionic ES100 and cationic ERL100 [86], where as the amount of cationic ERL100 increases, there is more anionic ES100 involved in the interaction, which leads to more and stronger physical entanglements, and in turn a decrease in fiber diameter. Since the emulsion ratio, drug content, and polymer used were all the same, only F2 was used as a representative. An enlarged SEM image of F2 fibers is shown in Figure 4e. Fibers F1–F4 all had smooth surfaces and were non-porous. The presence of drug crystals was not observed, giving a hint that the drug molecules were successfully loaded within the polymeric matrices in an amorphous state.

**Figure 5:**
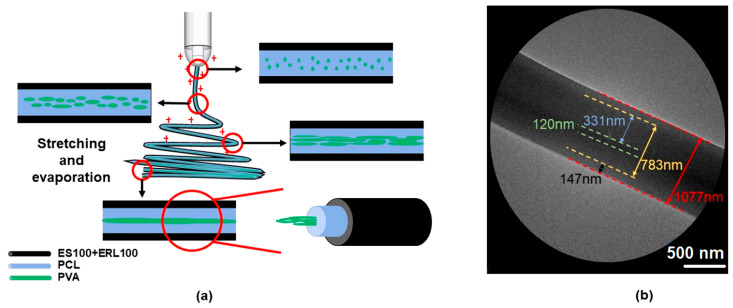
(**a**) Schematic diagram of the formation mechanism of bifluid electrospinning tertiary core–shell structure (**b**) TEM image of F2 fiber membrane. Red circles are enlarged to the images indicated by the arrows.

Figure 5a exhibits a schematic of the mechanism of tri-layer core–shell structure forming from the coaxial emulsion electrospinning. The core liquid emulsion undergoes stretching and evaporation-induced demulsification of the emulsion droplets under the action of an electric field force, and the innermost fiber morphology is gradually formed. Taking the F2 fiber as an example, the TEM evaluation is shown in Figure 5b. From the image, it can be seen that the fiber has a distinct tri-layer core–shell structure. The innermost layer of this tri-layer fiber is PVA and MTD which has a diameter of about 120 nm, and the middle layer is PCL and MTD with a thickness of about 331 nm. The total diameter of the core–shell fibers electrospun from the emulsion is about 783 nm. The shell layer consists of ES100 and ERL100 which is about 147 nm in thickness, and the diameter of the whole fiber is about 1077 nm. Electrospraying is a brother technique of electrospinning [87,88,89]; the present strategy and mechanism also give a hint for developing novel emulsion electrospraying processes in future.

### 3.3. Compatibility and Physical State Analysis of Electrospinning Fibers

#### 3.3.1. Compatibility between Fiber Components

**Figure 6:**
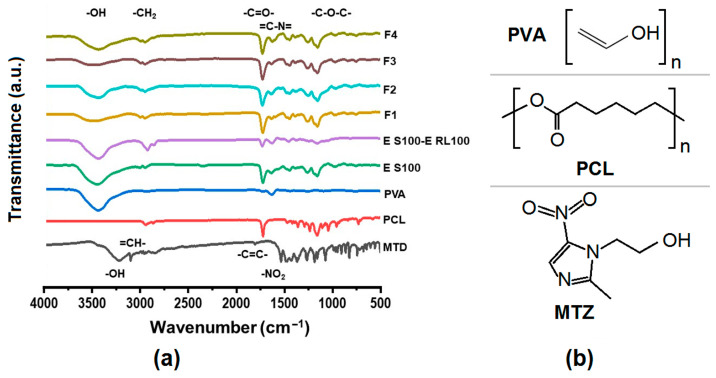
(**a**) FTIR of drug-loaded fibers and their raw materials, and molecular structures of PVA, PCL and MTD; (**b**) Molecular formula of PVA, PCL and MTZ.

The Fourier transform infrared spectroscopy (FTIR) spectra of the drug-loaded fibers and their raw materials as well as the molecular structure of the raw materials in the core solution are displayed in Figure 6a. MTD has a distinct peak of -OH vibration at 3218 cm^−1^, =CH- vibration at 3101 cm^−1^, -CH_2_- vibration at 2957 cm^−1^, -C=C- vibration at 1808 cm^−1^, and a characteristic peak of -NO_2_ vibration at 1536 cm^−1^. PCL has -CH_2_ vibration at 2944 cm^−1^, -C=O- vibration at 1723 cm^−1^, and symmetric and asymmetric -C-O-C- vibration at 1236 cm^−1^ and 1160 cm^−1^. PVA showed -OH vibration at 3441 cm^−1^. ES100 had -OH vibration at 3455 cm^−1^, -C=O- vibration at 1727 cm^−1^, and -C-O-C- vibration at 1161 cm^−1^. The drug-carrying fibers F1–F4 had -OH oscillations at about 3436 cm^−1^, -CH_2_ oscillations at about 2940 cm^−1^, -C=O- oscillations at 1730 cm^−1^, and -C-O-C- oscillations at 1160 cm^−1^.

In the FTIR spectral images, the presence of the characteristic peaks of the drug MTD was not found in the F1–F4 fibrous membranes. This indicates the formation of secondary interactions between the drug MTD and the polymers PCL and PVA. Combined with the schematic molecular structures of PCL, PVA and MTD in Figure 6b, it can be inferred that the MTD molecules (containing -OH groups) can act as proton donors, and the -C=O in the polymer PCL and the C=C in the PVA molecule can act as proton acceptors to form hydrogen bonds, resulting in the disappearance of characteristic peaks which appeared in the spectra of MTD raw powders. This indicates a good compatibility between the drug MTD and the polymeric matrices.

#### 3.3.2. Physical States of Components in Drug-Carrying Fibers

**Figure 7:**
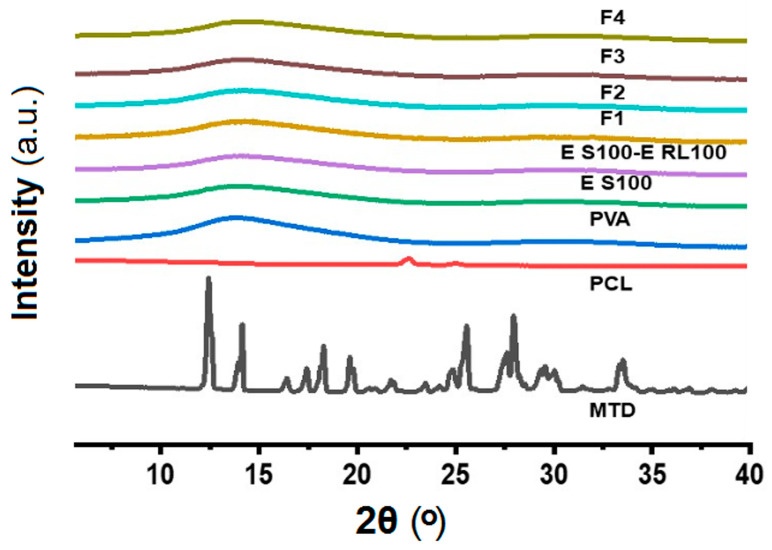
XRD patterns of drug-loaded fibers and their raw materials.

Figure 7 exhibits the X-ray diffraction (XRD) patterns of the drug-loaded fibers and their raw materials. From the figure, it can be significantly observed that the drug MTD has several obvious diffraction peaks at 2θ from 5° to 40°, which are 12.4°, 14.1°, 18.2°, 25.5°, and 27.9°, respectively, which indicates that the drug MTD has crystalline properties and pure MTD exists in crystalline form. PCL is also crystalline [90], but is semi-crystalline in nature, with a small peak at 2θ at 22.6°. The XRD images for PVA and other raw materials are amorphous, as indicated by the XRD images of PVA and other raw materials which are relatively smooth and have no obvious diffraction peaks.

The diffraction peaks of MTD and PCL were not detected in any of the F1–F4 fiber membranes in the XRD images. This may be due to a low drug loading in the fibers, which was only 2.78% (*w*/*w*) theoretically calculated by the solute concentrations in the working fluids and their flow rates. It could also be related to the chemical interaction between the antibiotics and PCL. On the one hand, the addition of antibiotics could decrease the crystallinity of PCL [91], as mentioned earlier by FTIR. On the other hand, MTD is dissolved in the organic phase together with PCL, and the transition from liquid to solid amorphous form is realized by electrospinning. In the electrospun polymeric composites, the fine compatibility between drug and polymer would prevent the possible formation of crystal nuclei and crystal growth. MTD is a poorly water-soluble drug. An amorphous state can benefit its dissolution and the realization of a designed drug controlled-release profile. Although the emulsion could improve the solubility of MTD to a certain extent [92,93,94], the drug loading was still small in this study. In future works, how to increase the drug loading will be investigated alone.

### 3.4. Analysis of Water Absorption and Wettability of Fiber Surface

**Figure 8:**
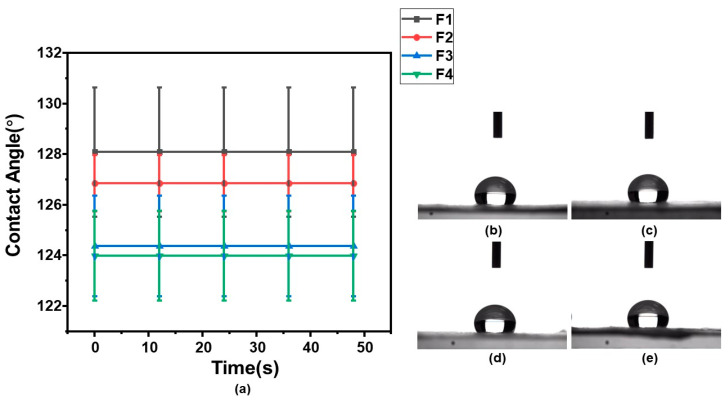
Water (hydrochloric acid buffer) contact angle measurements of fibrous membranes: (**a**) WCA change trends of fibrous membranes F1–F4; (**b**–**e**) Droplet images of fibrous membranes F1–F4 at 48th second.

Figure 8 displays the water (HBS, pH = 1.2) contact angles of F1–F4 membranes, demonstrating their acid and wetting resistance. From Figure 8a, the θ_S_ of F1–F4 were 128.1° ± 2.55°, 126.8° ± 1.11°, 124.4° ± 1.98°, and 123.9° ± 1.78°, respectively. It can be seen that the θ_S_ of fiber membranes F1–F4 decreased with the decrease in ES100 content, and θ_S_ did not change with time. Figure 8b–e show the droplets images on fibrous membranes F1–F4 at the 48th second, respectively. These results indicate that the WCA of the fibrous membranes did not change with time and was relatively stable overall.

In the water contact angle test, the samples are thought to be hydrophobic provided the θ_S_ > 90°. Here, the θ_S_ of F1–F4 were all greater than 90°, suggesting that they were hydrophobic fiber membranes. The fine acid-resistance performances resulted both from the hydrophobic properties of electrospun membranes and also the fact that the shell matrix ES100 only dissolves at a pH value of larger than 7.0 [95]. In addition, PCL is also a hydrophobic material in nature [96]. Therefore, although hydrophilic PVA was present as a drug carrier, the fiber membrane as a whole showed hydrophobicity.

### 3.5. Mechanical Properties Test

**Figure 9:**
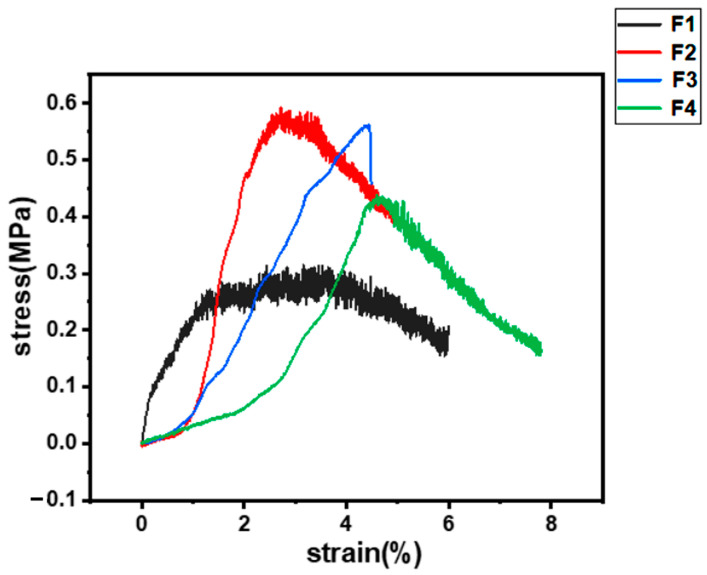
Stress–strain curve of fiber membrane.

Figure 9 shows the stress–strain curves of the F1–F4 fiber membranes, displaying the mechanical properties of the fiber membranes. As can be seen from the figure, the overall mechanical properties were inferior, the fiber membranes were brittle and not soft, and the tensile strengths were all less than 1 MPa. The F1 fibrous membrane with pure ES100 as the sheath fluid had the worst mechanical properties, only about 0.3 MPa. The mechanical properties of the fiber membranes were enhanced by the addition of ERL100, and the tensile strength of the F2 fiber membrane was the best among all the fiber membranes, but it was also only about 0.6 MPa. This may be related to the change in fiber diameter after the addition of ERL100. Studies have shown that fiber diameter and distribution affect the mechanical properties of fibers [97]. However, the tensile strength of the fiber film continued to decrease as the ERL100 content continued to increase and the tensile strain increased. The tensile strength of the F3 fiber membrane was approximately 0.56 MPa and the tensile strength of the F4 fiber membrane was approximately 0.44 MPa. This may be related to more anionic ES100 interacting with cationic ERL100 in the F3 and F4 fiber membranes.

### 3.6. Fiber Drug-Release Analysis

**Figure 10:**
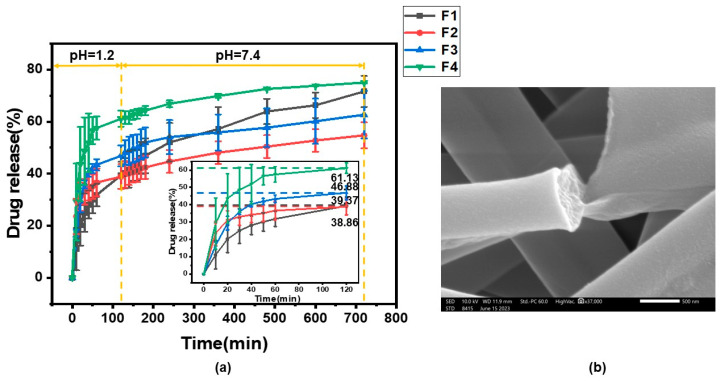
In vitro drug release: (**a**) fiber membrane drug-release curve; (**b**) SEM image of residue F2 after the exhaustion of loaded drug in the fibers.

Figure 10 displays the in vitro drug release from F1–F4 drug-loaded fiber membrane. As can be seen from Figure 10a, the drug release from F1–F4 fibrous membranes in the hydrochloric acid solution (HBS, pH = 1.2) during the first two hours of simulated gastric acid action was 39.37 ± 5.18%, 38.86 ± 4.77%, 46.88 ± 4.04%, and 61.13 ± 3.05%, respectively. Theoretically, the release of F1–F4 fiber membrane in HBS should not exceed 20%, but it actually exceeded 20%. On the one hand, it may have been due to the low flow rate of the emulsion containing the drug MTD, resulting in poor drug loading. On the other hand, it may be less effective in encapsulation. And obviously the drug release rates from the F3 and F4 fiber membranes were larger. It may be due to the fact that the acid resistance of the fiber membrane gradually deteriorated with the decrease in the content of pH-sensitive ES100, so the F4 fiber membrane released most of the drug in the HBS. Within 10 h of simulated intestinal fluid release, 32.24 ± 1.16%, 15.89 ± 0.21%, 15.79 ± 5.54%, and 13.89 ± 2.86% of F1–F4 fibrous membranes were released from the phosphate buffered solution (PBS, pH = 7.4), respectively. The release of F2–F4 was significantly lower. This may be related to the amount of ERL100 content added. On the one hand, ERL100 is insoluble in water, and an increase in the ionic strength of the medium will lead to a slower release rate. On the other hand, because of the addition of ERL100, the diameter of the fiber membrane decreases (as inferred from Section 2.2). Drug release behavior has been reported to be related to fiber diameter [98]. Therefore, F2–F4 fiber membranes showed a slower release rate in PBS than F1 fiber membranes. After 12 h of release, the total drug release from F1–F4 fiber membranes was 71.61 ± 5.95%, 54.75 ± 4.98%, 66.43 ± 10.45% and 75.03 ± 0.29%, respectively. This indicates that the drug-release behavior is directly related to the polymer composition of the fibers and also to the diameter of the fibers, which can be controlled by adjusting the polymer content. Figure 10b presents the SEM image which shows the fracture surface of the F2 fiber membrane after drug release.

To better understand the mechanism of drug release from core–shell fibers, four classical kinetic models were fitted to F1–F4 fibrous membranes to assess their release behavior, namely, the zero-order, first-order, Higuchi, and Ritger–Peppas kinetic models. Zero-order kinetic models are mainly used for controlled-release formulations that release at a constant rate, insoluble drug delivery in coated matrices and transdermal drug delivery [99,100]. First-order kinetic modeling is applied to delineate the extent of drug dissolution over time [101]. The Higuchi kinetic model is mainly utilized to study the kinetic process of drug release in controlled release systems and is the most widely used kinetic model [102]. In the Ritger–Peppas kinetic model, n represents the release index, which is assigned to indicate the mechanism of drug release. When n ≤ 0.45, drug release follows Fickian diffusion; when 0.45 < n < 0.9, the release mechanism follows non-Fickian diffusion (i.e., a combination of erosive and Fickian diffusion); and when n ≥ 0.9, drug release follows erosive diffusion [103]. In the development of fiber drug delivery systems, the clarification of drug-release kinetics can provide new ideas, in addition to overcoming the problem of blood concentration caused by the traditional drug delivery system [104,105,106].

**Figure 11:**
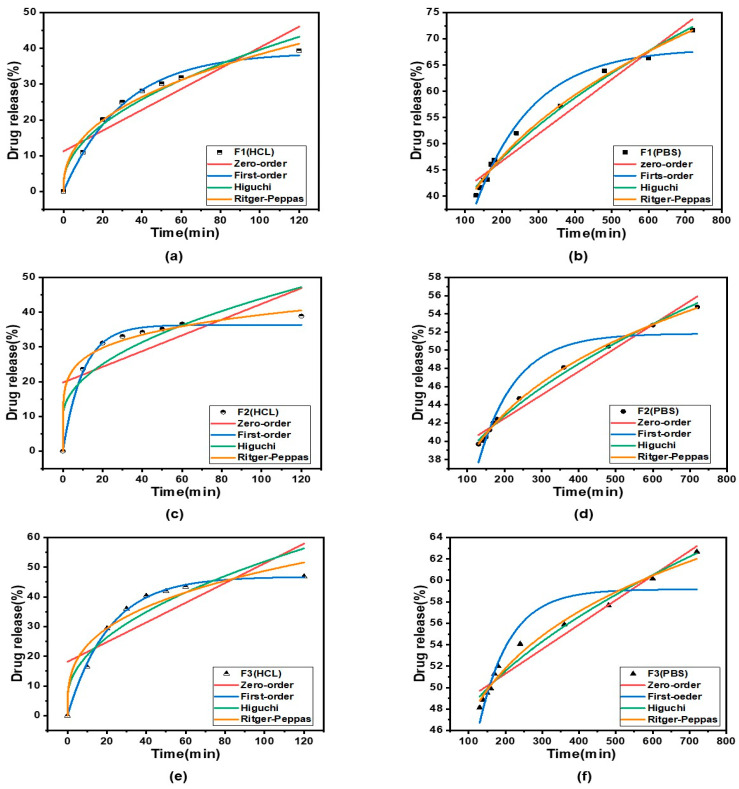

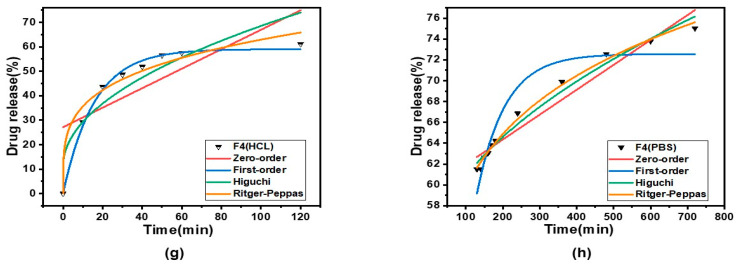
Kinetic curves (**a**,**c**,**e**,**g**) show the release kinetics of F1–F4 in HBS and (**b**,**d**,**f**,**h**) show the release kinetics of F1–F4 in PBS, respectively.

The kinetic equations for the in vitro release of F1–F4 are shown in Table 2, and the kinetic curves are shown in Figure 11. From the fitted equations and curves, we can ascertain that the n-values of Ritger–Peppas kinetic fittings for F1–F4 fibrous membranes in HBS were 0.41, 0.17, 0.31, and 0.25, which were smaller than 0.45, respectively, suggesting that the drug was released by Fickian diffusion in HBS. In PBS, the n-values of Ritger–Peppas kinetic fits for F1–F4 fiber membranes were 0.32, 0.19, 0.14, and 0.12, respectively, which were also smaller than 0.45, indicating that the drug MTD was also released by Fickian diffusion in PBS.

## 4. Conclusions

In the present study, multiple-polymer ESR/PCL/PVA tri-layer core–shell drug-loaded fiber membranes were successfully prepared by coaxial electrospinning methods with emulsions as the core working fluids. The fibrous membranes were characterized and evaluated, and their release kinetics were investigated with a view to better applying them for specific drug delivery. The SEM images displayed fibers that were homogeneous and smooth, and the TEM images confirmed that the fibers possessed an obvious tri-layer core–shell structure. FTIR and XRD showed that the drug MTD in an amorphous form was successfully loaded in the fibers. The F2 fiber membrane was the best performer among the four types of fibrous membranes. Its mechanical properties are outstanding compared to than the others. The tri-layer fibrous membrane released the drug in the buffer with a stable fibrous structure, in which the F2 fibers showed good acid resistance in HBS and extended release in PBS. The kinetic analysis of drug release showed that the F1–F4 fibers followed the Fickian diffusion law in both HBS buffer and PBS buffer. The tri-layer fibers have good hydrophobicity, which can better protect hydrophilic PVA and drugs in the inner section. The drug-loaded fiber membrane was prepared by dissolving the insoluble drug MTD in the emulsion. Coaxial emulsion electrospinning was able to create tri-layer core–shell nanofibers. In future, the capability of electrospinning in producing advanced functional nanofibers would further expand through its strong capability of tailoring components and compositions and their spatial distributions in a single-step and straightforward manner. Meanwhile, some directly related topics that deserve further investigation include: (1) the electrical field-induced assembly or phase separation mechanism of emulsion droplets; (2) how to elevate the drug release percentage in the specific regions of the gut; and (3) the demonstration of advantages of the tri-layer core–shell structures over the bi-layer core–shell and monolithic ones through in vivo experiments.

## Figures and Tables

**Table 1:** The composition and experimental parameters of electrospinning fibers.

Number	Shell	Core			Drug Content (MTD)	Flow Rate		Voltage
		PCL	PVA	PF-127		Shell	Core	
F1	15%ESR (100:0)	10%	8%	2%	2%	2.0 mL/h	0.5 mL/h	12.0 kV
F2	15%ESR (95:5)							
F3	15%ESR (90:10)							
F4	15%ESR (80:20)							

**Table 2:** Drug-release kinetics equations of fibers F1–F4 under different conditions.

Number	Model	HBS		PBS	
		Equation	R^2^	Equation	R^2^
F1	Zero-order kinetics	Q = 0.290 t + 11.203	0.7540	Q = 0.052 t + 36.248	0.9624
	First-order kinetics	Q = 38.810(1 − e^−0.033 t^)	0.9925	Q = 68.162(1 − e^−0.006 t^)	0.9637
	Higuchi kinetics	Q = 3.779 t^1/2^ + 1.833	0.9617	Q = 1.975 t^1/2^ + 19.282	0.9867
	Ritger–Peppas kinetics	Q = 5.889 t^0.41^	0.9758	Q = 8.689 t^0.32^	0.9914
F2	Zero-order kinetics	Q = 0.227 t + 19.721	0.4573	Q = 0.026 t + 37.335	0.9727
	First-order kinetics	Q = 36.314(1 − e^−0.098 t^)	0.9891	Q = 51.824(1 − e^−0.010 t^)	0.9062
	Higuchi kinetics	Q = 3.416 t^1/2^ + 9.769	0.7828	Q = 0.977 t^1/2^ + 28.952	0.9941
	Ritger–Peppas kinetics	Q = 17.691 t^0.17^	0.9867	Q = 15.999 t^0.19^	0.9987
F3	Zero-order kinetics	Q = 0.332 t + 18.118	0.6014	Q = 0.023 t + 46.722	0.9551
	First-order kinetics	Q = 46.786(1 − e^−0.047 t^)	0.9982	Q = 59.164(1 − e^−0.012 t^)	0.8842
	Higuchi kinetics	Q = 4.623 t^1/2^ + 5.677	0.8805	Q = 0.866 t^1/2^ + 39.297	0.9754
	Ritger–Peppas kinetics	Q = 11.470 t^0.31^	0.9411	Q = 24.631 t^0.14^	0.9815
F4	Zero-order kinetics	Q = 0.398 t + 27.167	0.5451	Q = 0.024 t + 59.590	0.9441
	First-order kinetics	Q = 59.029(1 − e^−0.063 t^)	0.9942	Q = 72.559(1 − e^−0.013 t^)	0.8986
	Higuchi kinetics	Q = 5.736 t^1/2^ + 11.172	0.8532	Q = 0.910 t^1/2^ + 51.730	0.9788
	Ritger–Peppas kinetics	Q = 20.168 t^0.25^	0.9689	Q = 34.500 t^0.12^	0.9940

## Data Availability

The data supporting the findings of this manuscript are available from the corresponding authors upon reasonable request.
